# Acute Myeloid Leukemia: A “Head to Toe” Examination

**DOI:** 10.7759/cureus.8526

**Published:** 2020-06-09

**Authors:** Robert Calleja, Sohaip Kabashneh

**Affiliations:** 1 Emergency Medicine, Wayne State University, Detroit, USA; 2 Internal Medicine, Wayne State University/Detroit Medical Center, Detroit, USA

**Keywords:** comprehensive physical exam, acute myeloid leukemia, missed diagnosis

## Abstract

Acute myeloid leukemia (AML) is a hematologic malignancy that can affect all blood cell lineages, the presentation varies, and infection is a common complication. This case involves a patient initially presenting with a necrotic foot ulcer and leukocytosis, ultimately leading to a diagnosis of osteomyelitis. After establishing adequate source control with serial debridements and intravenous antibiotics, the patient developed some knee swelling. On repeat assessment, he was discovered to have lymphadenopathy, and workup revealed AML. As indicated by this case, though it appeared a clear-cut diagnosis of osteomyelitis, there was an underlying malignancy that would have potentially gone unnoticed due to incomplete clinical examination.

## Introduction

Acute myeloid leukemia (AML) is the most common acute leukemia in adults, accounting for 80% of cases in this group [1]. The incidence of AML increases with age, in those over the age of 65 years it approaches 12.2 cases per 100,000 population [2]. Patients present with a wide assortment of complaints, commonly a combination of leukocytosis and signs of bone marrow failure, such as anemia and thrombocytopenia. The World Health Organization (WHO) requires the presence of 20% or more blasts in the bone marrow or peripheral blood to diagnose acute leukemia. AML, in general, has a poor prognosis, and as much as 70% of patients 65 years or older will die within one year of the diagnosis [3].

Patients present to health care facilities with a wide assortment of complaints daily, and often, cases can appear to be clear-cut diagnoses. However, many disease states can have numerous presentations. This report will help illustrate that despite how likely a diagnosis may appear, it is of paramount importance to always perform a thorough examination and deeply explore all features of a patient’s presentation.

## Case presentation

**Figure 1:**
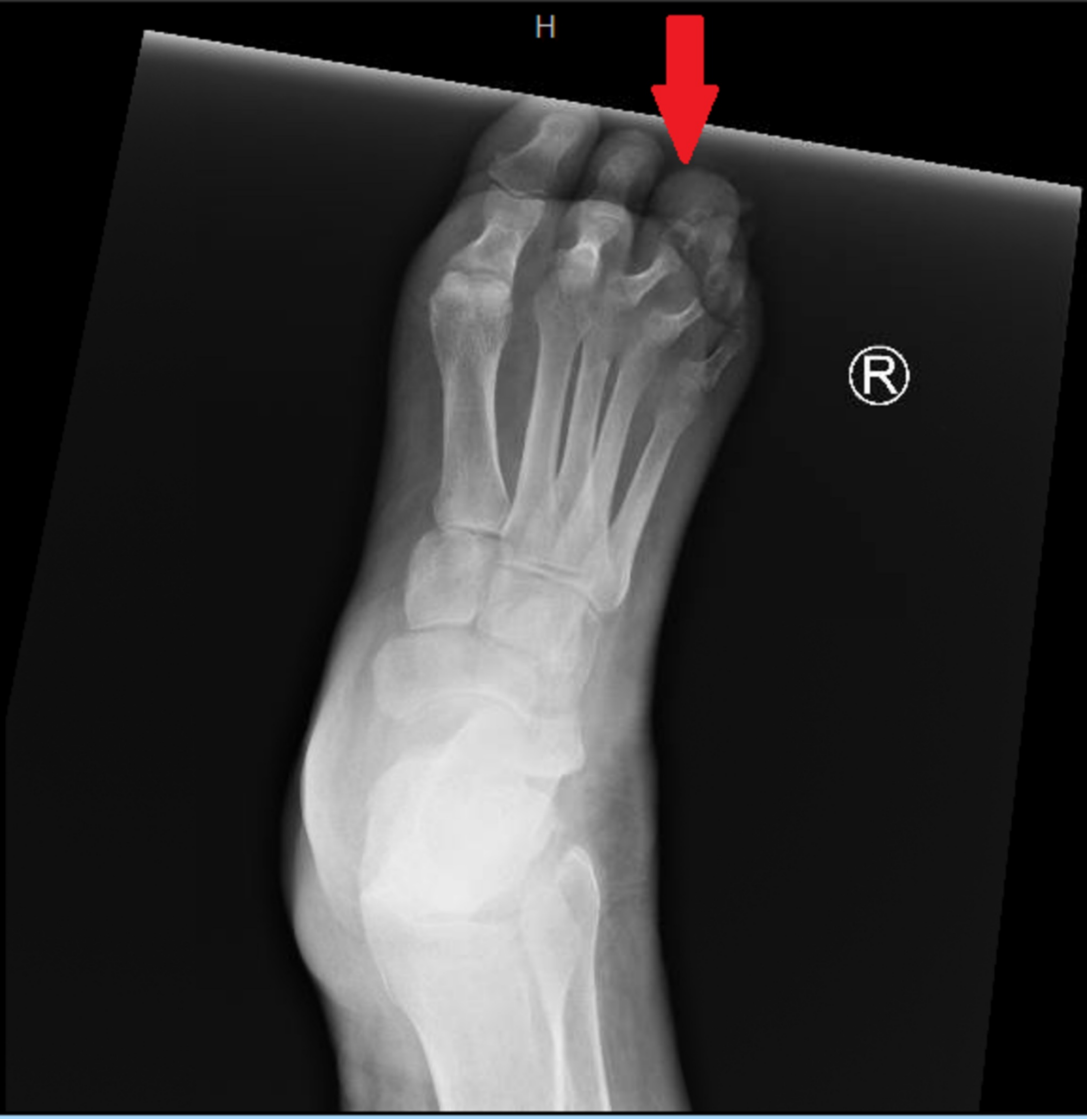
Anterior-posterior view of right foot illustrating a soft tissue swelling at the tip of the third toe.

**Figure 2:**
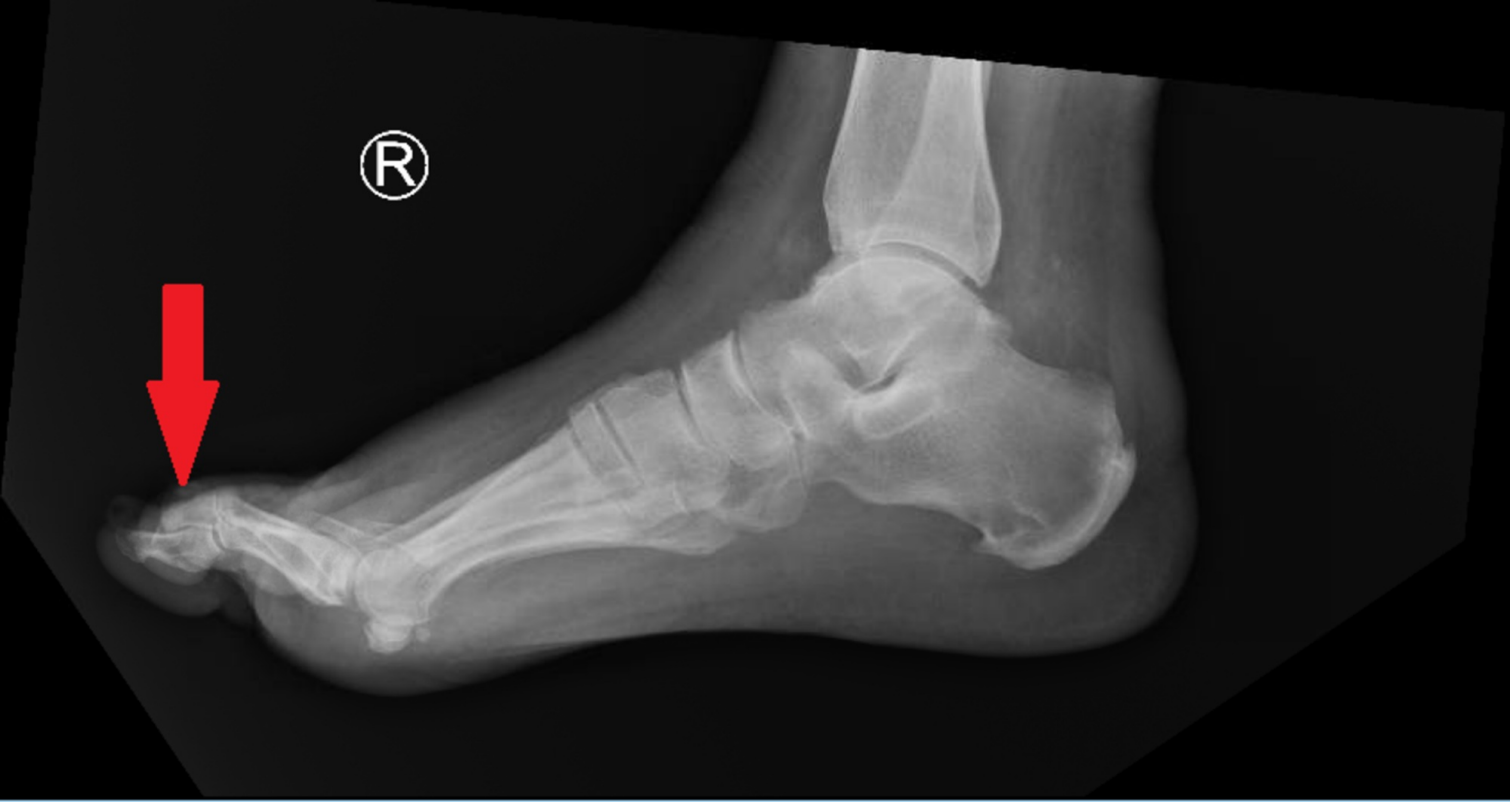
Lateral view of right foot illustrating a soft tissue swelling at the tip of the third toe.

We report a case of a 65-year-old African American man, with a past medical history of diabetes and gout. He presented with complaints of right third toe pain that began five days prior. He also reported constitutional symptoms, such as intermittent fever and chills, throughout the week. Two days prior to his arrival at the hospital, he reported his toe became discolored and had foul smelling drainage. Within the emergency department, he had a temperature of 36.4°C, a pulse of 84 beats per minute, a blood pressure of 125/76 mmHg, a respiratory rate of 18, and a pulse oximetry reading of 97% on room air. On physical exam, the heart and lungs exam was unremarkable, and his abdomen was soft and non-tender with no appreciable organomegaly. Cervical lymph nodes were not enlarged on the initial examination. Bilateral dorsalis pedis pulses were identified utilizing Doppler. The patient was found to have a non-tender third toe with purple/black discoloration. The toe was swollen and fluctuant with a distal wound that probed to the bone. Epicritic and gross sensations were diminished in both feet bilaterally. An X-ray was obtained, which illustrated that the tip of the distal phalanx of the third toe is missing. There was also soft tissue injury to the tip of the third toe (Figures 1, 2).

Initial laboratory studies indicated a white blood cell (WBC) count of 10.4 cells/µL (normal 3.5-10.6 cells/µL), differentials (neutrophils 2.7; lymphocytes 1.3; atypical lymphocytes 0.7; monocytes 4.8; eosinophils 0.1), a hemoglobin of 11.5 gm/dL (normal 13.3-17.1), a platelet count of 124 x 10^3/µL, C-reactive protein (CRP) of 139.9 mg/L (normal less than 10), and erythrocyte sedimentation rate (ESR) of 86 mm/hr (normal 1-13 mm/hr). Biochemical studies indicated glucose 183 mg/dL (normal 70-110), blood urea nitrogen (BUN) 21 mg/dL (normal 7-20), and creatinine 1.96 mg/dL (normal 0.70-1.30). Blood cultures were obtained on his first day of admission and indicated no growth after five days. Podiatric surgery was consulted, and a plan for surgery was made with the recommendation to hold antibiotics until after the operating room (OR).

The initial OR visit involved incision and drainage of a deep abscess in the right third toe with amputation at the metatarsophalangeal joint. Intraoperatively, the bone was found to be soft and gray, and cultures were obtained. The patient was started on empiric antibiotic therapy of aztreonam 2 g IV q8h and vancomycin 2,250 mg IV q36h until cultures were finalized. These bone and tissue cultures showed *Escherichia coli *and coagulase-negative staphylococci (CoNS), and his antibiotic regimen was eventually converted to unasyn 3 g IV and vancomycin 2 g IV, which was expected to continue in the outpatient setting for a total of six weeks of antibiotics. After the initial OR visit, MRI was obtained, which showed interval development of marrow edema, as well as enhancement in the second proximal phalanx and third metatarsal bones with adjacent soft tissue edema and enhancement, findings likely related to acute osteomyelitis. Throughout the first two weeks of his admission, he underwent serial debridements of the right foot with third metatarsal head biopsy, which indicated necrosis and acute osteomyelitis, ultimately requiring third metatarsal head resection.

Despite the establishment of adequate source control, the patient began experiencing new-onset right knee pain and worsening fatigue. At the time of developing knee pain, he denied complaints of fever, chills, diaphoresis, or night sweats. Examination of the right knee indicated effusion with tenderness to palpation and diminished range of motion. When asked about his knee pain, he stated he initially suspected the pain was associated with manipulation of the leg in his most recent OR visit two days prior. A more thorough examination revealed the presence of left submandibular lymphadenopathy and pale conjunctiva. No further lymphadenopathy was detected, and organomegaly was unable to be appreciated secondary to the patient’s body habitus. Slight right index finger effusion and decreased range of motion were also present in this examination. Initial concerns were for gouty versus septic arthritis, and therefore an arthrocentesis was performed. The fluid was turbid and milky in color with monosodium urate crystals present. There were 7,125 nucleated cells (77% neutrophil, 2% lymphocytes, 21% monocytes), and for red blood cells (RBCs) present. Fluid cultures were negative for growth. A uric acid level was found to be 9 mg/dL at this time.

Further inspection of serial laboratory studies that had been performed throughout his stay illustrated some concerning findings. On presentation, the patient had a hemoglobin of 10.1 gm/dL. This slowly drifted downwards throughout his hospital stay and was initially associated with acute blood loss anemia, secondary to his multiple OR visits. Hemoglobin stabilized around 7 gm/dL; however, the patient did require one transfusion when his hemoglobin reached 6.6 gm/dL. At admission, his platelet count started at 130 x 10^3/µL; however, this also trended down slowly throughout his hospital stay. The most concerning finding on the evaluation of his trending labs was the gradual rise in his WBC. At this point in his hospital stay, his WBC had reached levels as high as 58.5 cells/µL, despite a level of 10.4 cells/µL upon presentation. The initial rise in his WBC was associated with his operations and later attributed to steroid-induced leukocytosis when the patient was found to have an acute gout exacerbation. Further inspection of the WBC differential illustrated more concerning values, with a predominantly monocytic leukocytosis.

**Figure 3:**
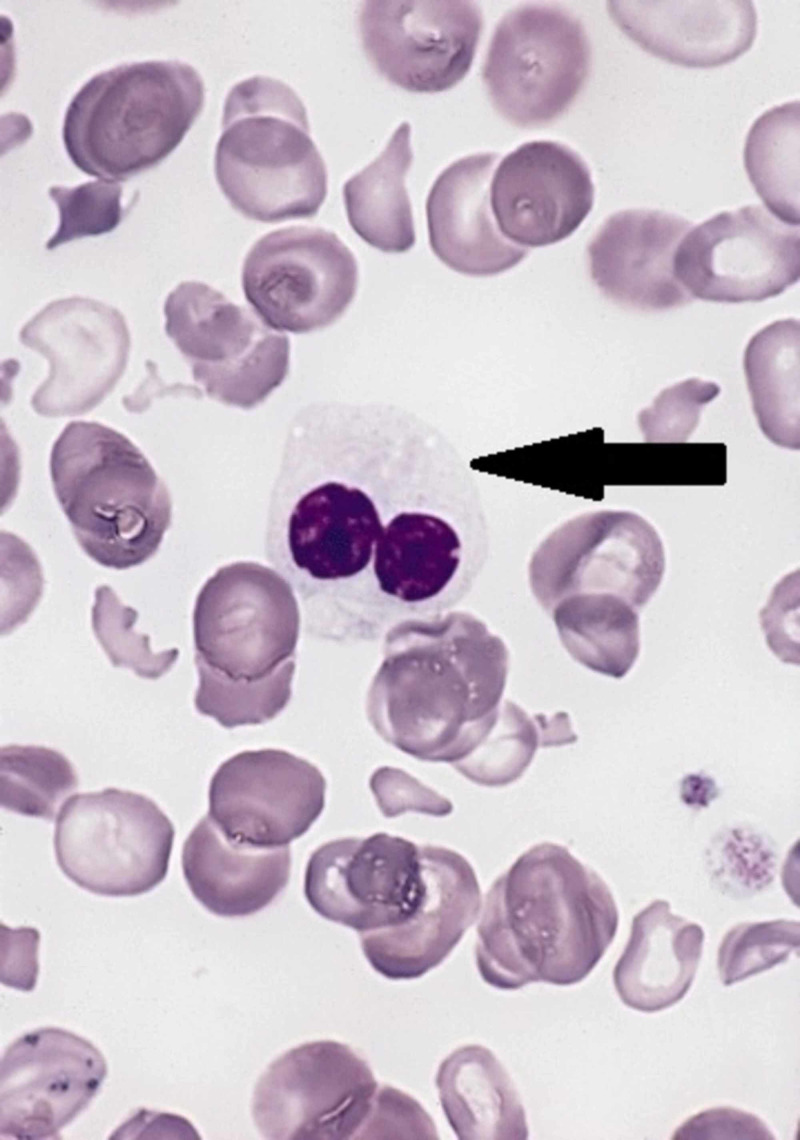
Example peripheral blood smear showing Pseudo Pelger-Huet bodies. Image component modified from The Armed Forces Institute of Pathology (AFIP) - PEIR Digital Library (Pathology image database). Image# 404814.

Due to these findings, the patient had a further workup performed, including a peripheral blood smear, bone marrow aspiration, and flow cytometry. The peripheral blood smear did indicate a few blasts with macrocytic normochromic anemia and thrombocytosis. Pseudo Pelger-Huet bodies were visualized within the peripheral blood smear, illustrating concerns for an underlying myeloproliferative process (Figure 3). A sternal bone marrow aspiration was performed given his body habitus and illustrated an elevation of the myeloid series with a leftward shift, predominantly with monocytes, as well as abnormal appearing eosinophils. Blast cells accounted for 22% of the cells seen. Results of the flow cytometry performed on this sample were also indicative of AML, illustrating an Inv(16), which is a favorable prognostic marker of AML [4]. Thus, with the repeated physical exam and further diagnostic workup, the patient was given a final diagnosis of AML. Following the stabilization of the patient’s current condition, he was transferred to an affiliated cancer institute for the management of his AML.

## Discussion

Ensuring that a thorough physical exam is performed and the corresponding studies are carefully investigated is paramount to patients’ health and outcomes. In this case, the patient appeared to have a presentation corresponding to a clear-cut diagnosis of a necrotic foot wound with underlying osteomyelitis in a patient with a history of diabetes mellitus. On deeper investigation, yielding cervical lymphadenopathy and the concerning laboratory studies, the presence of the underlying hematologic abnormalities was discovered. Incidences, such as this, seem to happen quite commonly. A prior study illustrated that missed diagnoses are present in approximately 29% of cases within hospitals [5]. These missed findings negatively affect patient outcomes, and it was hypothesized that earlier determination of these findings could have altered the management of these patients’ health [5]. It is important to address that a thorough physical exam is quintessential to the health and wellness of all patients. The correlation of in-depth patient history with a thorough physical exam helps to guide clinical management. Should findings within the examination be missed, the decision-making process may be shifted into a different direction, leading to inaccurate management of the patient. In fact, it was noted in one study that the association between an incomplete bedside assessment (both history and physical exam) and misdiagnosis attributed to 42% of incorrect diagnoses [6]. Therefore, to improve patient care, focusing on a comprehensive exam is necessary to improve clinical reasoning.

There appear to be two areas in which clinicians can improve their physical examination skills. Studies have found that hospitalists spend 17%-18% of their time regarding direct patient health care [7,8]. Clinicians have moved towards more indirect patient care focus, involving communication with other physicians and documentation. However, this removes opportunities for clinicians to find the key exam findings necessary to make the appropriate physical diagnosis. This can lead to delays in the detection of clinical findings, and as one study reported that in cases with missed or delayed diagnoses, 76% were due to physical examination inadequacies and lead to delay in management, on average of up to five days [9]. Therefore, a shift back towards direct patient care needs to occur to ensure diagnoses are not missed. Secondarily, continued education and training regarding the physical exam could ensure that clinicians are more confident in the utilization of their skills and could result in more thorough examinations. In a study comparing self-confidence in performing physical exam maneuvers and the utility of said maneuvers, determinations were made that students, residents, and interns alike do not have complete confidence in their exam skills. Hypotheses could lead one to extrapolate that a lack of self-confidence deters continued education of the future generations, leading to diminished diagnostic skills pertaining to the physical exam [10]. To overcome these lapses of confidence, institutions could reinforce the teaching model, which allows the medical community to cultivate their physical exam skills. This could assist health care providers with the confidence in utilizing these skills in practice and avoiding the overlooking of certain, more subtle physical exam findings.

Additionally, it is important to effectively assess the laboratory findings associated with physical exams. While sometimes laboratory studies may appear to be appropriate for the current circumstances, the correlation with the physical exam can discover serious pathology that may otherwise be overlooked. Such as in this case, though there appeared to be a clear explanation for the initial rise of leukocytosis, by correlating this appropriately with the physical exam, a serious underlying pathology was discovered. Therefore, to ensure optimal patient care in the future, these adjustments should be adopted by all health care providers.

## Conclusions

On presentation, patients may appear to be clear-cut diagnoses. However, a comprehensive clinical assessment is essential to shed light on other, possibly serious problems that patients may harbor. Missed diagnoses are common within hospitals, which can be explained by inadequate time spent in direct patient health care and the trend to depend on extensive investigations by health care providers.
